# “Many old people taking care of old people”: Experiences of older adults after Hurricane María in Puerto Rico

**DOI:** 10.1371/journal.pone.0316156

**Published:** 2025-01-16

**Authors:** Adrián J. Santiago-Santiago, Jeffrey Ramos-Santiago, Eliut Rivera-Segarra

**Affiliations:** 1 School of Behavioral and Brain Sciences, Ponce Health Sciences University, Ponce, Puerto Rico; 2 Department of Public Health Sciences, University of Rochester Medical Center, Rochester, New York, United States of America; Wuhan Mental Health Centre, CHINA

## Abstract

The Puerto Rican population has presented demographic changes resulting in a greater proportion of older adults than almost any other country in the world, with an estimated 28% of the total population being over 60 years of age. A key public health issue in Puerto Rico (PR) is older adults’ mental health and wellbeing. Located in the Caribbean, PR is prone to natural hazards such as hurricanes, which are a known threat to older adults’ overall health and wellbeing. However, the needs of older adults in PR and their mental health have been largely neglected, especially in less visible and marginalized rural areas. Therefore, the main objective of this study is to examine the challenges and needs of older adults following Hurricane María in the rural towns of Adjuntas and Castañer, PR. We implemented an exploratory qualitative research design informed by the principles of Liming methodology. We used semi-structured interviews to gather data and analyzed it using thematic analysis. A total of 25 participants engaged in our interview process including older adults (n = 15) and community caregivers and leaders that provide services to older adults (n = 10). We present three main themes regarding older adults’ experiences after Hurricane María: 1) Challenges to wellbeing (i.e, loneliness, lack of resources), 2) Relational solidarity (i.e., communal support, equitable resource sharing) and 3) *La brega* (i.e., adaption and *autogestión*). Older adults in Adjuntas and Castañer addressed the emergent challenges after Hurricane María such as loneliness and lack of resources by relying on their communities. These findings highlight the role of community support as a key component to understand and foster older adults’ wellbeing following a natural hazard.

## Introduction

Puerto Rico’s (PR) population has presented demographic changes resulting in a greater proportion of older adults than almost any other country in the world [1]. It is estimated that 28% of the total population is over 60 years of age, while 6% is over 80 [2]. PR is a non-incorporated territory of the United States (US) subjected to a colonial relationship [3] and is home to almost 3.2 million US citizens who mainly speak Spanish [2]. However, although the older adult population seems somewhat similar to other high-income countries such as Japan (28.2%), Italy (22.8%) and Finland (21.9%) [4], PR faces some unique challenges. The recent economic and social environment in PR (i.e., government debt and economic recession, political corruption, suboptimal healthcare services) as well as multiple natural hazards (i.e., hurricanes, earthquakes and the COVID pandemic) have been a cause of increased public health concern regarding their impact on older adults’ health and wellbeing, particularly their mental health [5–8].

Mental health problems can be common among older adults, hampering their overall health and wellbeing [9]. For instance, older adults have been found to experience higher rates of suicide, loneliness and social isolation compared to the general population [9,10]. Additionally, a previous literature review has documented health disparities among ethnic minority older adults (e.g. Latinos) such as higher rates of depression and stress, lower quality of life, sleep problems and poor healthcare access compared to whites [11]. These disparities are mainly attributed to little-considered cultural factors such as the importance of family-embedded issues and differences in mental distress expression (i.e., somatization, pain, headaches, gastrointestinal distress) in Latinos [11]. Literature has also shown that mental health promoting factors such as resilience, coping behaviors, and community engagement are higher among older adults and fundamental for understanding and promoting health and wellbeing among this population. Despite this, their psychological needs (e.g., cognitive impairments, social isolation, economic and decision-making stressors) are often unaddressed or poorly managed across healthcare settings because of the lack of personnel aware of these concerning issues [12,13]. Additionally, the socio-cultural, historical, and political contexts in which older adults live may also impact their health, development, and coping processes [11]. Thus, it is key to examine older adults’ mental health in diverse social and cultural contexts such as PR.

A major cause of psychological distress are natural hazards (i.e., hurricanes, earthquakes, pandemics, etc.), which have and will continue to increase in intensity and frequency due to climate change [5,14,15]. Natural hazards have been shown to be linked to poorer mental health outcomes. For example, exposure to natural disasters such as hurricanes, floods or earthquakes can increase the risk of post-traumatic stress disorder, major depression, or generalized anxiety [16–18]. Furthermore, severity and continuity over time of exposure to natural hazards may lead to worsened mental and physical health outcomes [19]. Recent evidence suggests that older adults are almost 2 times more likely to develop PTSD symptoms and adjustment disorders following a natural hazard when compared to younger adults [20]. Moreover, older adults who live in rural areas are at a greater risk of experiencing adverse situations during and after disasters due to their complex medical needs and long geographical distances from resources [21]. Past research also indicates that systemic factors such as bureaucratic procedures, limited livelihood options for older persons, and prioritization of younger versus older persons could drive older adults’ risks after natural hazards [22]. Arguably, these were some of the same realities that older adults in PR had to contend with after the impact of Hurricane María.

### Hurricane María in PR

As an archipelago located in the Caribbean, PR is highly prone to natural hazards, particularly hurricanes. Hurricane María was a category 4 hurricane that landed on September 20^th^, 2017, and became one of the most catastrophic hurricanes to hit PR and the United States, resulting in 2,975 estimated deaths [23,24]. Some authors have estimated a total of 1,205 excess deaths, of which 1,038 were among people aged 60 years or older [25]. PR had already experienced another hurricane just two weeks before María, Hurricane Irma, and together they caused severe damage to the power grid, roads, communication networks, food, and health services [26]. It has been estimated that Hurricane María caused up to $90 billion in damages, making it one of the costliest storms in the US since 1900 [27]. The damages caused frequent power outages and the overall recovery lasted up to six months, although it took much longer in some areas of the island, namely rural communities [28].

These events drew attention to PR’s long history of environmental injustice and degradation, community disparities, slow response and inadequate preparedness, and the archipelago’s high susceptibility to cyclones due to a combination of geographic, economic, and political problems [15,29,30]. However, little is known about the experiences and perspectives of older adults living in rural communities after the hurricane, and their needs and actions have remained largely invisible. Two areas in PR located right in the heart of PR’s rural area are Adjuntas and Castañer. These communities are recognized for their high number of older adults living in poverty but also their unique and well-established history of social and community work [31–34].

### Context and study objective

**Fig 1:**
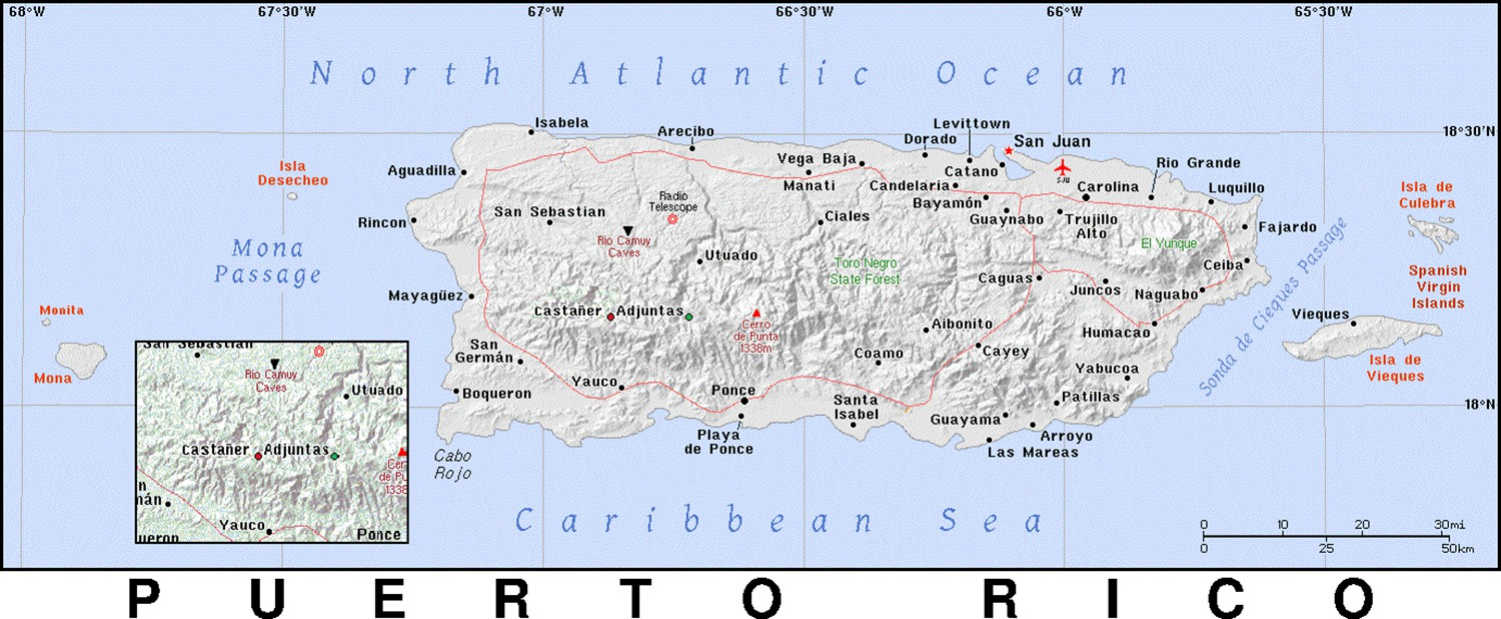
Map of Adjuntas and Castañer.

This research was conducted in the rural and centermost region of PR, in the municipality of Adjuntas and Castañer (See Fig 1). Adjuntas is a town in PR with a population of approximately 18,100 inhabitants [33]. It is estimated that almost 67.1% of the population lives below the US federal poverty line. Furthermore, out of the total population, 21% are older adults (65 and over) [33]. On the other hand, Castañer is known as a *poblado* (smaller town or village-like, without an official designation). It borders Adjuntas and other municipalities with the highest levels of poverty and older adults in PR (i.e., Lares: 24% 65 or older, 51% below poverty line; Maricao: 30% 65 or older, 45% below poverty line; and Yauco: 21% 65 or older; 42% below poverty line) [35–37]. This predominantly coffee growing region has recently gained increased recognition worldwide for its unique community organizing approaches and services [32,34], As a result, older adults in Adjuntas and Castañer often turn to community organizations and service providers for support after natural hazards [31].

On the other hand, previous post-hazard literature in PR has focused on the quality of life and natural hazard preparedness of older adults, and political planning forth wellbeing and management of chronic diseases of older individuals after a natural hazard [38,39]. Nonetheless, little research has explored the mental health needs of older adults in PR in the aftermath of a disaster, and much less in rural regions [40]. This is of vital importance in the context of post-disaster research in PR as data has evidenced a sharp increase in mental illnesses in the aftermath of Hurricane María [40]. Therefore, given their unique history and needs, the main objective of this study is to examine the experiences and perceptions of older adults, as well as community leaders and caregivers who provide care to the elderly, about the challenges and needs that older adults have had after Hurricane María in the rural towns of Adjuntas and Castañer, PR.

## Materials and methods

### Study design

Given the exploratory nature of this study, we implemented a qualitative research design [41]. We conducted this study by following the Consolidated Criteria for Reporting Qualitative Research (COREQ), a checklist developed to improve transparency and reliability in qualitative research [42] (See S1 Text). Recruitment was then completed using purposive sampling. We conducted individual in-depth semi-structured interviews among two main groups who experienced Hurricane María in the municipality of Adjuntas and Castañer: 1) older adults (aged >60 years or older) and 2) community leaders and caregivers engaged in any type of care (i.e., social support, healthcare, etc.) for the older adult population, in order to gain a deeper understanding of the experiences of older adults during and after Hurricane María.

Additionally, this work was informed by the principles of Liming methodology, a culturally affirming research approach that draws from the cultural practice of liming (an event common in the Caribbean in which people come together to relax, share food or music and talk, but also for communicating and sense-making) [43]. This Caribbean-centric methodology is used to affirm the ways of knowing and the worldviews of communities and to enable both researchers and participants to be active agents of knowledge construction [44]. It is important to mention that, even though we did not consider this methodology a priori, we implemented key elements pertaining to it as the study moved along. For example, although initially designed to implement semi-structured individual interviews lasting no more than 60-minutes, we immediately noticed this approach was limiting for this context and population. When participants invited us to their homes to share their experiences after Hurricane María, a common element across most interviews was the offering of coffee to begin the conversations. The sharing of this beverage immediately turned the conversation into a more familiar one in which interviewees and interviewers were able to communicate and make sense of the Hurricane María event. This led interviews to last longer than initially proposed. Guided by Liming methodology, interviewers engaged in humor, mutual storytelling about personal experiences with Hurricane María, extended interview times, and food consumption as practices to facilitate the interview process with older adults [43]. We believe this flexible approach allowed us to better connect, share experiences and co-construct meaning with participants in this context.

### Reflexivity statement

Our research team was composed of young adult men and women (less than 35 years old) all of which lived in PR during Hurricane María. Members of our team were students in a PhD program in Clinical Psychology at the time of the study, supervised by a faculty member (ER-S). All authors have old adult family members (i.e., grandmothers, grandfathers, mothers, fathers, etc.) that lived through Hurricane María and are currently living in PR. Furthermore, two of the authors are from Adjuntas (ER-S) and Castañer (AJS-S), and one continues to live in Castañer to this date (AJS-S). Having lived in the region, the first and third authors were acquainted with key community-based organizations (i.e., Casa Pueblo and Hospital General Castañer), and some participants recognized them (ER-S) or their family members as members of the Adjuntas and Castañer communities. Therefore, authors conveniently identified community leaders who then became community partners supporting our study. This project is the result of our individual and family experiences as well as our contextual firsthand knowledge and agency as researchers living in PR. Our position as researchers studying the communities we come from and still live in, shapes our engagement, understanding and interpretation of finding in several ways. One example is when a small pilot funding opportunity became available, the author’s (ER-S) lived experiences in this context before and after the event played a key role in the selection of the topic, population and study location. Additionally, the completion of the project was determined by the first author (AJS-S) and supported by the second author (JR-S). Finally, although these experiences and positionality shape our engagement in this study, we want to highlight that we do not aim to speak for or in the name of older adults in PR.

### Participants

A total of 25 participants engaged in our interview process including older adults (n = 15) and caregivers and community leaders that provide formal and informal care services to older adults (n = 10). The inclusion criteria applied to the first group were: 1) being older than 60 years of age; and 2) having experienced Hurricane Irma and María in Adjuntas and/or Castañer. For the service providers group, the inclusion criteria consisted of 1) being older than 21 years of age, 2) engaged in any type of care (i.e., social support, healthcare, etc.) for the older adult population, 3) having experienced Hurricane Irma and María in Adjuntas and/or Castañer. Participants varied in their marital status, achieved academic degree and income, although most older adults described themselves below the poverty line. Refer to Tables 1 and 2 for a detailed summary of the participants’ socio-demographic information.

**Table 1:** Socio-demographic data of older adults.

Variable	Frequency	%
Gender		
Men	7	47%
Women	8	53%
Marital status		
Single	1	7%
Married	8	53%
Living with partner	2	13%
Widow	4	27%
Academic grade reached		
Less than high school	7	47%
High School	5	33%
Bachelors	2	13%
Doctoral	1	17%
Monthly income		
≤ $500	7	50%
$501-$999	1	7%
$1,000-$1,999	3	21%
$2,000-$2,999	2	14%
$4,000-$4,999	1	7%

Note: n = 22 Missing values = 3.

**Table 2:** Socio-demographic data of community leaders and caregivers.

Variable	Frequency	%
Gender		
Men	1	14%
Women	6	86%
Marital status		
Single	4	57%
Married	3	43%
Academic grade reached		
Associate	1	14%
Bachelors	3	43%
Doctoral	3	43%
Monthly income		
≤ $501-$999	1	13%
$2,000-$2,999	2	33%
$3,000-$3,999	1	17%
$4000-$4,999	1	17%
$5,000+	1	17%

Note: n = 22 Missing values = 3.

### Procedure

The research protocol for this study was approved by the Ponce Health Sciences University Institutional Review Board (IRB-Protocol 1903007163). Following IRB approval and using a snowball sampling procedure, we began the recruitment process. We first contacted our previously identified community partners (i.e., community leaders from Casa Pueblo and Hospital General Castañer) who supported outreach efforts by the team (i.e., using promotional flyers and word of mouth). To facilitate recruitment, our community partners recommended potential participants based on eligibility criteria. Furthermore, individuals who were interested in the study such as older adults, family members of older adults, community and organizational leaders and caregivers, among others, also approached the research team to participate. This process was seamlessly reproduced by study participants who also identified potential candidates and provided them with the team’s contact information. After explaining the research study, additional questions were clarified, and the individual interview was coordinated at the participant’s preferred date and location.

The semi-structured interview guide consisted of the following themes: 1) sociodemographic information, 2) general information regarding personal experiences after Hurricane María, 3) perceived emotional and social wellbeing before and after Hurricane María, and 4) perceived impact of physical and social environment changes after Hurricane María (See S2 Text). All participants gave written informed consent to take part in our study. No potential participant refused to participate in the study and no participant dropped out of the study. Most older adult participants requested that interviews be conducted in their own homes. Most community and organizational members preferred their respective work offices. However, despite the selected interview spaces, the team ensured that no one else was present during the interviews, although some occasional interruptions during the interviews did occur (i.e., phone ringing and knocking on doors). All interviews were conducted in Spanish. Participants in this study received a $25 compensation. Although all participants were provided a stipend, it is important to highlight that at the end of the interviews, some participants initially mentioned that the stipend was not necessary and emphasized their ethical responsibility to share what they know even without compensation. Interviews were audio-recorded and conducted face-to-face during the period of March 29, 2019, to October 30, 2019, by the second (JR-S) and last (ER-S) authors. Interviews generally lasted between an hour and a half to two hours. However, interviews with older adults frequently lasted more, usually about two and a half hours and in some instances more.

To maintain rigor and validity, the last author (ER-S) met with the interviewer (JR-S) for a debriefing after each individual interview to address issues such as: underemphasized areas, specific techniques to manage the interviewer’s emotions, among others. After debriefings, the interviewer proceeded to conduct the next scheduled interview. This process continued until we achieved data saturation [45]. Transcripts were not returned to participants for comments or corrections.

### Data analysis

All audio-recorded interviews were transcribed verbatim in word processors and analyzed. We used thematic analysis to approach participant data and further organize it, which allowed us to interrogate and interpret patterns found in the dataset [46]. No qualitative data analysis software was used in this study. Rather, the team conducted an open coding process directly on the transcripts in the word processor. Furthermore, based on inductive analysis [47], the team developed a codebook that captured older adults’ disaster experiences after Hurricane María (e.g., solidarity, mental health). As such, we carried out an inductive analysis process by extrapolating the codes and themes based on participants’ quotes. First, the first and second author (AJS-S, JR-S) read the dataset repeatedly to familiarize themselves with the data and develop a list of preliminary codes. These two authors then took on the role of coders and independently assigned preliminary codes to all the interviews. During initial coding, memos and comments were also written in the transcripts to reflect and improve themes, to distinguish between the perception of the research team and that of the participants, and to minimize bias in the interpretation of meaning. After preliminary coding, both coders met with the third author (ER-S) who served as a mediator to integrate different perspectives and ensured agreement regarding the codes that were used.

Based on the most salient patterns identified, coders made a second review of the codes to develop the initial themes and select the representing quotations. These were brought back to the entire team (AJS-S, JR-S, ER-S) for discussion, to engage in a reflective process, and to ensure consensus. During the first review, emerging themes were related to challenges faced by older adults after Hurricane María, as well as their use of general resources as coping strategies. During the second review, the team identified more specific themes about relational solidarity and *la brega* among older adults. After reaching consensus, the final themes and their respective quotations were determined. These are presented in the results section below. The authors translated all quotes to English for publication purposes. Original Spanish quotes are included as supplementary material. We attempted to maintain the original meaning of the quotations and included some words in Spanish that we considered had no literal equivalent in English.

## Results

In light of the unique challenges faced by older adults in Adjuntas and Castañer, the results presented here highlight three main emerging themes about the experiences of older adults after Hurricane María in PR: 1) Challenges to wellbeing, 2) Relational solidarity, and 3) *La brega*. Furthermore, despite our inclusion of Hurricane Irma as a natural hazard relevant to our study, it is worth noting that participants’ narratives mostly focused on Hurricane María. For this reason, the themes and quotes presented from now on will only highlight the experiences lived after the passage of Hurricane María specifically. The results presented are based on the triangulation of the narratives of the older adult participants and the community leaders and caregivers. In Table 3, we present three definitions of each theme as framed by study participants.

**Table 3:** List of themes.

Themes	Descriptions			
**Challenges to wellbeing** **Relational solidarity** ***La brega***	This theme reflects the stressors affecting the overall ability of older adults to feel positive about their lives and living conditions after hurricane María. It includes quotes focusing on caring for other old people while being old themselves, feelings of loneliness and refusing to leave their homes. It refers to a reciprocal commitment and engagement by older adults and community members to address their threats and needs after Hurricane María. This definition stems from past literature on relational solidarity [48]. Quotes included here reflect community resource sharing, a pride and sense of belonging and challenges to maintain solidarity. This theme describes a self-managed individual and collective initiative to accept and work hard in spite of the limiting and limited resources and conditions. This definition stems from past literature on the Puerto Rican phenomenon of the same name [49]. In includes quotes focused on examples of working with less, work as self-motivation and commitment to the community.			

### “Many old people taking care of old people”—Challenges to wellbeing after a natural hazard among older adults in Adjuntas and Castañer, PR

For participants [15/15], the hurricane brought many demands and lack of resources that became challenges for their psychological health and emotional functioning. Due to the broad demographic of older adults in Adjuntas and Castañer, having to care for other older adults in their communities had become a common reality for the elderly. However, caring for other elderly became much harder after the hurricane as the few young relatives they had left migrated away in search of work and safety, and would rarely visit. As such, multiple participants [12/15] lacked the means and access to care and had to rely on neighbors and older relatives to care for each other. This challenge became so common that providers [10/10] who serve the elderly in the region consider it a public health problem. When we asked about his professional experience with the topic, one of the interviewees told us:

“There are many old people taking care of old people, I mean… there are many, when they do not have adequate resources, an old person taking care of another old person is not the best thing…” (Professional Administrator of local hospice program).

While living in fragile and lonely environments, participants [10/15] had to care for others while juggling their own chronic and comorbid health conditions, such as Alzheimer’s, diabetes, and dementia, to name a few. Some [3/15] even said they had to care for bedridden relatives and neighbors while lacking the strength to lift them or help them move. An elderly caregiver we interviewed was a vivid example of this experience. In a mountain residence up winding roads and far from urban centers and medical services, this caregiver’s home awaited. From her empty house, she had to care for her bedridden husband, alone and without access to utilities. When we asked her to describe her experience, she said:

“This is very difficult, well… and then just being alone here, well, with… Not entirely alone because my brother is here, but he tends to go out and so on. But this, to live such a situation alone. Washing clothes by hand, with a bedridden person, you know, that’s been very difficult.” (Female denizen, 71 years old; wife and caretaker).

Due to the infrequency of family visits, participants [12/15] felt significant loneliness following Hurricane María. Older participants supported their children and grandchildren in their search for more favorable living opportunities, but still felt forgotten and neglected. Providers [10/10] realized their older clients felt alone but noticed their loneliness had worsened after the hurricane. Providers even noticed that older adults were coming to appointments looking for company and conversation, rather than for their own health needs. One of the providers we interviewed, who was also an older adult from Adjuntas, indicated that she saw this loneliness among her clients often. In her interview, she told us:

“… they live alone (Older adults), you know, the children, the family, they forgot, they left, and these people live alone.” (Female denizen, 60 years old, Alzheimer’s patient caretaker)

On top of this loneliness, hurricane-force winds and heavy rains had led the homes of the elderly to suffer severe structural damage. Some participants [14/15] tried to fix their homes but struggled to recover due to financial insecurity and lack of resources, leaving them with the emotional strain and powerlessness of a frustrated desire. A recently widowed, elderly participant was one such case. Sitting in his rocking chair on his balcony, he welcomed us into his newly built wooden home and explained why Hurricane Maria was so difficult for his family:

“[B]ecause we lost practically everything we had. The roof and everything flew out, everything went flying and that’s traumatizing. So, since you are not wealthy, you do not have money, you have to wait for donations…” (Male denizen, 68 years old).

Recognizing the loneliness and the risks of their damaged homes, younger relatives tried to get their elderly out to have them close and support them. However, many participants [7/15] were reluctant to leave and preferred to stay despite the challenges they faced. For them, there was no other option than to stay. In general, the interviewed older adults experienced constant grief and loneliness after the hurricane because of their pervasive challenges. So much so that when we asked one of the providers about the sadness he saw among his older patients, he said:

“I can tell you that most of who lived through that experience are no longer here. They already died in the program. And many of those were because of sadness, they were very sad.” (Social Worker for local hospice).

### “In this community each one was working together”—relational solidarity among older adults in Adjuntas and Castañer

To cope with these challenges, many participants [10/15] turned to their neighbors for support and exchanged resources reciprocally. From the first day after the hurricane, participants saw that their neighbors were experiencing similar needs and decided to share their supplies despite having their own shortcomings. With the little they had, older adults gave resources (e.g., water, food, shared their power generators) to their neighbors without expecting anything in return. Soon after, older adults in the region joined together as a community to assist each other through manual labor. The elderly came together to clean roads and remove debris on their own with old tools they found in their sheds. They even went to stores on foot to get supplies for their communities whenever a neighbor could not go out. Beyond attending to their material needs, the elderly neighbors kept each other company and nourished each other with conversation, which helped them feel less alone. Taken together, these acts were living examples of relational solidarity, a concept of community respect and support to reach greater collective wellbeing [48]. For instance, when we asked one of the older participants about her relationship with her community, she said:

“… the commitment that we had as citizens of here of Castañer. The union, the commitment…the familiarity because everyone here practically, although we were not family, since we were all always familiar with what do you need, if I go someplace to buy something its “what do I bring to you”, “what do I give to you”. That. Mainly among everything was the union we had, but the commitment. To be aware of everyone…” (Female Caregiver feminine, 61 years old)

These acts of solidarity evoked strong feelings of belonginess in their communities [8/15], which became a source of strength for older adults in Adjuntas and Castañer. This feeling for their communities soon turned into pride in themselves since the older adults felt they were working hard despite the challenges they faced. One older participant felt honored to be part of a small neighborhood in Adjuntas who organized itself to attend community needs without help from the local government. She said:

“…I’m proud to belong to Portillo, right, to Tanamá…” (female denizen, 60 years old, caregiver of Alzheimer patients).

Although she lived alone, participants like her found comfort in the people around her and their willingness to share. These reciprocal aids became a symbol of belonging and companionship that allowed older adults to cope with their loneliness and sparse family support. One occasion, lacking help even motivated them to act. For example, when asked about help from the local government, an older participant told us:

“…[m]ovement from the government was very slow and that forced people to come together to work, everyone in their respective area.” (Male denizen, 68 years old).

The participants suffered due to the lack of solidarity from the local government, especially from the mayors of Adjuntas and Lares. The government was offering aid at the time; however, help was concentrated far from these mountain communities. Most older adults [13/15] felt forgotten when they heard that the government was sending essentials to certain regions, but nothing was reaching them. One of the older participants said:

“It took about four months after Hurricane María, if not more, for a mayor to come here. No one from the government showed up here. Nothing.” (Female caregiver, 61 years, old)

Nevertheless, the elderly were not the only ones who showed relational solidarity in Adjuntas and Castañer. Community-based and service-providing institutions [7/10] mobilized support to remote regions with elderly in need. In the absence of government assistance, local organizations such as Castañer General Hospital and Casa Pueblo became pillars of support for older adults in the region. To help hard-to-reach communities, an interviewed provider said:

“We formed a team of doctors, mental health, and nursing staff, and we began to visit shelters. It could be said that we had the tools to know what was going to be done there, because there was no plan.” (Castañer male clinical psychologist)

Similarly, non-profit organizations, local churches of different denominations, and different institutions strengthened these efforts to bring food and support to the elderly. When we asked one of the participants what kind of help she had received, she said:

“The Red Cross, the National Guard did come a lot, many people came here personally and one of these came from institutions, they also came. And many people here still bring things to this date.” (Female denizen, 71 years old)

According to this interviewee, aid continued to benefit older adults in the region even a year and a half after Hurricane María, which is when we conducted the interviews. In other words, relational solidarity continued to be a source of support even long after the hurricane. However, some participants [5/15] saw neglect where others saw solidarity. A few interviewees indicated that no one from their communities helped them or checked on them, not even their closest neighbors. These participants reported that their neighbors were apathetic and individualistic during the recovery process. For example, one participant stated:

“It was difficult… But the indifference here in Adjuntas is also difficult… Nobody, nobody here…I’ve been here for about 39 years more or less and my neighbors, some came later, but there were some who had been here for a long time… [and were] quite indifferent…” (Female denizen, 80 years old)

In summary, although a few older adults had to address their challenges alone, most participants benefited from a sense of solidarity and belonging that allowed them to nurture their emotional wellbeing. As one interviewee succinctly summarized:

“…in this community, they were well united. In this community, each one was working together.” (Female denizen, 78 years old).

To cope with the challenges, this sense of solidarity amidst disaster recovery also came with a sense of hard work which we will elaborate in the next section.

### “If I decay, I am not going to solve anything…”—*La brega* among older adults in Adjuntas and Castañer

Despite the solidarity that benefited participants, many older adults still struggled to manage their challenges. Although support was helpful, some benefited more than others. As a result, many older adults [12/15] organically engaged in *la brega* (i.e., a Puerto Rican term loosely similar to “the hustle”). *La brega* refers to a practice of radically accepting precarious conditions in a given situation and working through despite the limitations. For example, many participants had to work with the fewest resources available, such as their small homes made of wood and metal panels. From the perspective of *la brega*, the suboptimal conditions of the house mattered little. The house was still useful if it was standing. Despite living in vulnerable spaces, participants prioritized making makeshift fixes to their homes and moving on to the next achievable goal. When talking about the support he received from his wife, one of the older participants said:

“The whole house [was] destroyed, and… she told me; ‘We are alive, we have to push forward’. And my wife, who is strong, gave me her support. She would always tell me; ‘we are going to get through it [*bregar*], forget about it, little by little’. And my roof had plastic coverings for more than a year…” (Male denizen, 68 years old)

For this participant, being reminded that there were things more important than material losses offered the support he needed after the hurricane. Similarly, other participants [13/15] made the most of the few materials they could find by bartering with their neighbors, which helped them adapt to scarcity and loneliness. According to participants, the goal of *la brega* seemed to be resourcefulness. When we asked one of the interviewees what materials she used to cope with the shortages, she said:

“…I would look for a candle, lit it, and with that we would light up around. And to cook, since we couldn’t, well we would go downstairs too. If we ran out of gas, we would go down there to the kitchen, turn on a little fire over there and yes, [I] worked through [*bregaba*] downstairs, cooking and everything.” (Female denizen, 80 years old)

Despite the emotional decline brought on by their limited conditions, older adults empowered and motivated themselves to get back up. This self-motivation allowed them to maintain their daily functioning and move their recovery along. Participants [13/15] felt they had to drive themselves and drew strength from any possible source to avoid stagnating. For instance, one participant said:

“So, well, how do I put it, I am human, like, I do not give up, I am not insensitive, but I remain firm, but always seeing and visualizing that if I decay, I’m not going to solve anything, that I need to draw strength from myself, strength from wherever I have to look for it, to face whatever comes and contribute what I can.” (Female denizen, 78 years old).

Interviewed providers [5/10] also used *la brega* in their organizations to offer support services to older adults in the region. As such, providers felt they had a shared responsibility to their communities. Speaking about how they engaged with community work, one provider said:

“…Well, because you have the people, because you have the infrastructure, because you have the relationships, because you have, in the case of Casa Pueblo it has credibility and a history. When we face problems, we face them [with our] heads up high and we solve them with what’s within our reach, with the limitations that one might have." (Casa Pueblo-Community-based organization from Adjuntas)

Similarly, another provider shared this feeling of responsibility towards her community members. When asked how she felt about her work after María, one provider said:

“Since I quickly got involved in working, maybe I didn’t have a lot of time to regret and not because I wasn’t emotionally upset, it was just time to get involved, to do what I had to do.” (Female psychologist)

Overall, older adults in Adjuntas and Castañer used *la brega* to encourage themselves, face adversity, and exercise their social responsibility as community members. At the same time, providers served older adults using *la brega* to support them despite having their own limitations. Similar to relational solidarity, *la brega* was another meaningful strategy that older adults used to confront their challenges and nurture their emotional wellbeing following Hurricane María. In Table 4, we present a data table with the specific number of older adult participants and community leaders and providers that endorsed each narrative and examples.

**Table 4:** Number of older adult participants and community providers who endorsed theme narrative and examples.

Themes	Older adults (n = 15)	Community leaders and service providers (n = 10)
**Challenges to wellbeing** Lack of access to care Caring for family members Caring for bedridden relatives Having physical health conditions Feelings of loneliness Lack of resources Refusal to leave home **Relational solidarity** Turning to neighbors for support Belongingness and pride Help from community institutions Help from non-profit organization and churches Community indifference Lack of government solidarity ***La brega*** Working through limitations Adaptation to scarcity Self-motivation Organizations sharing responsibility	15 12 3 10 12 14 7 10 8 10 10 5 13 12 13 13 4	10 4 0 10 10 8 4 5 4 7 4 0 4 5 5 3 5

## Discussion

The main objective of our study was to examine the experiences of older adults, as well as community leaders and caregivers who provide care to the elderly, about the challenges and needs that older adults had after Hurricane María in the rural towns of Adjuntas and Castañer, PR. We conducted in-depth semi structured qualitative interviews and further analyzed the data using thematic analysis guidelines to identify participants’ main emerging themes. Overall, older adult participants in our study experienced challenges to their wellbeing such as lack of resources and feelings of loneliness. Participants navigated these challenges through relational solidarity as the community turned to support and equitable resource-sharing. Even more so, amidst the adversities, older adult participants in our study engaged in *la brega* as a measure to motivate themselves and adapt to scarcity.

Firstly, older adults in Adjuntas and Castañer came face-to-face with many adversities during Hurricane María. Through our interactions with the participants, we discovered multiple challenges at the individual (e.g., lack of resources), community (e.g., loneliness due to family absence, lack of younger caretakers), and organizational level (e.g., negligible governmental support). No notable differences were observed between the experiences of participants in Adjuntas when compared to Castañer, but rather their narratives aligned and pointed to common experiences. Although the identified needs were primarily individual, they imply an underlying relational or community quality that is inseparable from these individual experiences. Case in point, older adults seemed to turn both to themselves and their immediate community to cope with the distress experienced after natural hazards. Our results are consistent with previous literature establishing that neighborhood and social ties are important to maintain mental wellbeing after a natural hazard [50]. In spite of their challenges, it is important to highlight how older adults in Adjuntas and Castañer took individual experiences such as their prevalent lack of access to essentials and converted them into more communal efforts, such as a network to reciprocally share resources with their neighbors. These actions empowered older adults in Adjuntas and Castañer to self-manage their disaster-related challenges through community support and mutual aid, which past research has suggested to be an important component of empowerment for older populations [51]. These community-based strategies also support prior literature that dictates that a lack of access to close relationships can be detrimental to the psychological wellbeing of older adults [52]. These solidary actions, whether from older adults or caregivers, point to survivor ethics, a concept which represents how individuals engage in a pro social response after facing a hazard firsthand [53]. In this sense, participants in our study seemed to share the experience of a community-wise and general moral recovery after the impact of the hurricane.

On the other hand, research has found that mental health generally tends to decline among older adults as they reach older ages [54]. Although we did not explore the mental health of older adults across different periods of their lives, our results seem to show that our participants were able to use their community-based coping strategies to manage their emotional distress effectively, even the oldest participants. This is particularly true in terms of *la brega*, which mainly concerns a communal practice where many people come together to *bregar* (“attend") a situation and gather the necessary resources to manage an emergency [49]. These results seem to suggest that older adults in Adjuntas and Castañer have reliable strategies to maintain their mental wellbeing despite their older age. This is also consistent with prior studies that found that leaning toward different resources simultaneously, particularly social relationships and community networks, are key to improving wellbeing and recovering after natural hazards, even more so amongst older adults living in rural areas [55–57]. Moreover, these community self-organizing practices that older adults used after Hurricane María may seem to share a similarity to resilience, as authors define it as the capacity and availability to adapt when facing adversity [31]. Nonetheless, contrary to resilience, *la brega* suggests a community-level attitude to handle situations of daily living despite a marked inequality of power, which has developed in the face of the violent and constantly changing conditions of a deep colonial history, which is characteristic of Puerto Rican identity [58].

Moreover, other researchers predicted that natural hazards like Hurricane María would lead to a mass migration of the general population [59], yet our participants did not appear to have considered leaving their homes as an option, both before and after the storm. Rather, many held on to their houses and communities despite their families reaching out and insisting for them to move to arguably safer geographical spaces. Although this finding implies a greater risk of loneliness, the persistence to stay echoes a stronger place attachment to their living spaces, in addition to their resistance to migrate among older adults compared to younger adults [60], consistent with the reality of a predominantly older adult population in Adjuntas and Castañer. Moreover, we would argue that being part of Adjuntas and Castañer seems to be a more significant part of the identity of older adults when compared to younger ones, which could be contributing to their persistence to stay. This coincides with factors related to emotional connection, such as local culture (e.g., language), memories associated with the homeland, and a sense of family and community that are in tune with place attachment and which hinders migration from PR [61].

To address the distress of older adults after a natural hazard, it is also important to look at the needs that were not met during the 2017 event. Past literature highlights the significance of minimizing most post-disaster negative experiences and providing continuous mental health support during and after natural hazards [62]. Even though our results indicated that community organizations and private sectors came together to assist the population, these efforts were insufficient, namely because of the government’s notable absence in the support process. However, these issues must be considered beyond the individual level. Rather than putting emphasis on the hurricane in isolation, the particular context and history of PR must be prioritized as part of disaster preparedness and response [62], especially the specific context of small rural communities with high levels of poverty.

So far, the severe impact of the hurricane can be understood as a consequence of the colonial experiences (i.e., lack of government action due to economic and political influences) Puerto Ricans have endured for many decades [63,64], which have also affected older adults in the island. Furthermore, rural areas such as Adjuntas and Castañer tend to be socioeconomically disadvantaged because they are geographically distant from the larger cities in PR (e.g., San Juan), and therefore, older adults living in these regions tend to suffer the most because they are often unable to practice healthy lifestyles and behaviors [65]. This reality puts into perspective the level of attention this specific population demands from governmental and private sectors. Future research should explore the experiences and shortages of other vulnerable populations (i.e., socioeconomically disadvantaged populations) in distant geographical locations after other natural hazards such as hurricanes, earthquakes, among others [55]. Policymakers and local social service providers should emphasize public health measures to assist these rural and elderly populations, particularly after natural hazards. For instance, special attention could be brought to measures such as “Law 14–1996: Special Law for the Development of Castañer”, which is a public policy to ensure the social, economic and cultural development of Castañer and surrounding neighborhoods [66]. Although the local government passed this law to guarantee greater wellbeing by offering economic incentives, we consider that its implementation has not taken into account the needs and precarious conditions that this region tends to experience after a natural hazard. Along with that, older adults caring for older adults, particularly after hazards, are often unaware of assistance services available to them such as non-medical homecare services, medical adult day programs, transportation and meal delivery services, and legal, financial, individual, and family counseling [67]. Efforts should be made to educate and promote these services to the older adult population. Moreover, as a prevention measure, public policies should increase initiatives to provide support services for older people living in rural and isolated areas. For example, accompaniment services could be provided through home visits, and particularly the establishment of community-run support and shelter centers could be established in hard-to-reach areas for older adults to visit, especially if they are in need during an emergency or after a natural hazard. Particularly, older adult populations and community organizations in the region could benefit from an implementation of the aforementioned laws and public aid measures that are more grounded in the historical damage that these events have recently brought.

### Strengths and limitations

One of the strengths of our study is the expansion of knowledge of the remote and isolated geographical regions of Adjuntas and Castañer. This study focused on increasing the visibility of the experiences of underserved populations (i.e., older adults living in poor conditions) in particularly hard to access places. Furthermore, because Castañer has no specific designation, being a *Poblado* surrounded by four towns, its older adult population could be considered even more underserved and invisible. Additionally, some of our team members grew up in Adjuntas and Castañer, and had firsthand experience navigating these community spaces. This representation by the team facilitated access to the sample and, in turn, served to better contextualize the experiences of the participants.

On the other hand, our study has some limitations worth mentioning. Firstly, we began data collection one year after the impact of Hurricane María. As such, the study’s timing represents a limitation since the passage of time could lead to recall bias. Additionally, all participants from Castañer were from central areas of the *Poblado* and the experiences of older adults and caregivers from lesser-known areas of Castañer were excluded. Future studies on Castañer should include participants from lesser-known areas to account for their specific experiences.

## Conclusion

This study aimed to explore the mental health needs and wellbeing of older adults during and after a natural hazard in a particularly rural context, inside a territory with a long history of colonial oppression and neglect. Previous research had already emphasized the significance of improving government emergency response measures as it decreases risk of trauma and distress, as well as safeguards the mental health of older adults [62]. Nonetheless, older adults in Adjuntas and Castañer seem to lack public support and often rely on themselves and their communities to face natural hazards on the island. Older adults also seem to adhere to the relational solidarity of their communities and to *la brega* to manage their challenges and maintain their wellbeing during difficult emergencies. Taking these results into consideration, it is important to support community-based programs and local initiatives in the development and implementation of policy and direct services as they may represent one of the most far-reaching and effective resources for older adults in the geographical and sociopolitical context of Adjuntas and Castañer.

## Supporting information

S1 Text COREQ checklist. (DOCX)

S2 Text Semi-structured interview guide. (DOCX)

S3 Text Translated quotes. (DOCX)

## References

[1] Matos-Moreno A, Verdery AM, Mendes de Leon CF, De Jesús-Monge VM, Santos-Lozada AR. Aging and the Left Behind: Puerto Rico and Its Unconventional Rapid Aging. Meeks S, editor. Gerontologist. 2022 Jun 13; 62(7):964–73. doi: 10.1093/geront/gnac082 35696667 PMC9372893

[2] census.gov. Quick Facts Puerto Rico [Internet]. U.S. Census Bureau; 2021 [cited 2023 Sep 18]. Available from: https://www.census.gov/quickfacts/fact/table/PR/LFE041220#LFE041220.

[3] Cheatham A, Roy D. Puerto Rico: A U.S. Territory in Crisis [Internet]. Council on Foreign Relations; 2022 [cited 2023 Sep 18]. Available from: https://www.cfr.org/backgrounder/puerto-rico-us-territory-crisis.

[4] prb.org. Countries With the Oldest Populations in the World [Internet]. Population Reference Bureau; 2022 [cited 2023 Sep 18]. Available from: https://www.prb.org/resources/countries-with-the-oldest-populations-in-the-world/.

[5] Puttick S, Bosher L, Chmutina K. Disasters Are Not Natural. Teach Geogr 2018 Jan 7; 43(3): 118–20. https://www.jstor.org/stable/26538739.

[6] Hernández AR. In graying Puerto Rico, the elderly face climate disasters alone. [Internet]. The Washington Post; 2023 [cited 2023 Sep 18]. Available from: https://www.washingtonpost.com/nation/2023/01/13/puerto-rico-hurricanes-climate-elderly/.

[7] Parés Arroyo M. En riesgo los adultos mayores que viven en instituciones de cuido por el alza de COVID-19 [Internet]. El Nuevo Día; 2023 [cited 2023 Sep 18]. Available from: https://www.elnuevodia.com/noticias/locales/notas/en-riesgo-los-adultos-mayores-que-viven-en-instituciones-de-cuido-por-el-alza-de-covid-19/.

[8] Tormos-Aponte F, Prudencio W, Painter MA, Franklin B. Clientelism and Corruption in the Wake of Disasters. [Internet] CENTRO; 2022 [cited 2023 Sep 18]. Available from: https://centropr.hunter.cuny.edu/publications/clientelism-and-corruption-in-the-wake-of-disasters/.

[9] Donovan NJ, Blazer D. Social Isolation and Loneliness in Older Adults: Review and Commentary of a National Academies Report. Am J Geriatr Psychiatry. 2020 Aug; 28(12):1233–44. doi: 10.1016/j.jagp.2020.08.005 32919873 PMC7437541

[10] Troya IM, Babatunde O, Polidano K, Bartlam B, McCloskey E, Dikomitis L, et al. Self-harm in older adults: Systematic review. Br J Psychiatry. 2019; 214(4), 186–200. doi: 10.1192/bjp.2019.11 30789112

[11] Jimenez DE, Park M, Rosen D, hui Joo J, Garza DM, Weinstein ER, et al. Centering Culture in Mental Health: Differences in Diagnosis, Treatment, and Access to Care Among Older People of Color. Am J Geriatr Psychiatry. 2022 Jul; 30(11):1234–51. doi: 10.1016/j.jagp.2022.07.001 35914985 PMC9799260

[12] Albert I, Kornadt AE. The Corona Pandemic and Its Implications for the Mental Health and Mental Healthcare of Older Adults. GeroPsych. 2022 Mar 1; 35(1):1–3. 10.1024/1662-9647/a000286.

[13] Carpenter BD, Gatz M, Smyer MA. Mental health and aging in the 2020s. Am Psychol. 2021 Dec 30; 77(4):538–50. doi: 10.1037/amp0000873 34968089

[14] Guggenheim M. Introduction: Disasters as Politics–Politics as Disasters. Sociol Rev. 2014 Jun; 62(1):1–16. 10.1111/1467-954X.12121.

[15] Hayward RA, Morris Z, Otero Ramos Y, Silva Díaz A. “Todo ha sido a pulmón”: Community organizing after disaster in Puerto Rico. J Community Pract. 2019 Jul 29; 27(3–4):249–59. 10.1080/10705422.2019.1649776.

[16] Lieberman-Cribbin W, Liu B, Schneider S, Schwartz R, Taioli E. Self-Reported and FEMA Flood Exposure Assessment after Hurricane Sandy: Assoc with Ment Health Outco. PLoS One. 2017 Jan 27; 12(1). 10.1371/journal.pone.0170965. PMC5271356 28129410

[17] Ruggiero KJ, Gros K, McCauley JL, Resnick HS, Morgan M, Kilpatrick DG, et al. Mental Health Outcomes Among Adults in Galveston and Chambers Counties After Hurricane Ike. Disaster Med Public Health Prep. 2012 Mar; 6(1):26–32. doi: 10.1001/dmp.2012.7 22490934 PMC3951740

[18] Schwind JS, Norman SA, Brown R, Frances RH, Koss E, Karmacharya D, et al. Association Between Earthquake Exposures and Mental Health Outcomes in Phulpingdanda Village After the 2015 Nepal Earthquakes. Community Ment Health J. 2019 May 17; 55(7):1103–13. doi: 10.1007/s10597-019-00404-w 31102165

[19] Shultz JM, Galea S. Preparing for the Next Harvey, Irma, or Maria—Addressing Research Gaps. N Engl J Med. 2017 Nov 9; 377(19):1804–6. doi: 10.1056/NEJMp1712854 29019711

[20] Parker G, Lie D, Siskind DJ, Martin-Khan M, Raphael B, Crompton D, et al. Mental health implications for older adults after natural disasters–a systematic review and meta-analysis. Int Psychogeriatr. 2015 Jul 27; 28(1):11–20. doi: 10.1017/S1041610215001210 26212132

[21] Ashida S, Robinson EL, Gay J, Ramirez M. Motivating rural older residents to prepare for disasters: moving beyond personal benefits. Ageing Soc. 2015 Aug 20; 36(10):2117–40. doi: 10.1017/S0144686X15000914 30013285 PMC6045947

[22] Joseph J, Jaswal S. Elderly and Disaster Mental Health: Understanding Older Persons’ Vulnerability and Psychosocial Well-Being Two Years after Tsunami. Ageing Int. 2020 Jul 1; 10.1007/s12126-020-09375-w.

[23] nhc.noaa.gov. Hurricane MARIA [Internet]. National Hurricane Center and Central Pacific Hurricane Center; 2022 [cited 2023 Sep 23]. Available from: https://www.nhc.noaa.gov/archive/2017/al15/al152017.update.09201034.shtml.

[24] Santos-Burgoa C, Goldman A, Andrade E, Barrett N, Colon-Ramos U, Edberg M, et al. Ascertainment of the Estimated Excess Mortality from Hurricane Maria in Puerto Rico [Internet]. Global Health Faculty Publications; 2022 [cited 2023 Sep 18] Available from: https://hsrc.himmelfarb.gwu.edu/sphhs_global_facpubs/288.

[25] Cruz-Cano R, Mead EL. Causes of Excess Deaths in Puerto Rico After Hurricane Maria: A Time-Series Estimation. AJPH. 2019 Jun 5; 109(7): 1050–52. doi: 10.2105/AJPH.2019.305015 30998411 PMC6603484

[26] Minemyer P. Damage from hurricane María pushes medical services in Puerto Rico to the brink [Internet]. Fierce Healthcare; 2017 [cited 2023 Sep 23] Available from: https://www.fiercehealthcare.com/population-health/hurricane-María-puerto-rico-medical-services-natural-disaster-harvey-irma.

[27] National Hurricane Center. Costliest U.S. tropical cyclones table updated. [Internet]. National Oceanic and Atmospheric Administration; 2018. [cited 2024 May 29] Available from: https://www.nhc.noaa.gov/news/UpdatedCostliest.pdf.

[28] Shultz JM, Kossin JP, Shepherd JM, Ransdell JM, Walshe R, Kelman I, et al. Risks, Health Consequences, and Response Challenges for Small-Island-Based Populations: Observations From the 2017 Atlantic Hurricane Season. Disaster Med Public Health Prep. 2018 Apr 6; 13(1):5–17. doi: 10.1017/dmp.2018.28 29622053

[29] Dietrich A, Garriga-López M, Garriga-López C. Hurricane Maria Exposes Puerto Rico’s Stark Environmental and Health Inequalities [Internet]. Social Science Research Council: Insights from the Social Sciences; 2017 [cited 2023 Sep 23] Available from: https://items.ssrc.org/just-environments/hurricane-maria-exposes-puerto-ricos-stark-environmental-and-health-inequalities/.

[30] Padilla M, Rodríguez-Madera SL, Varas-Díaz N, Grove K, Rivera S, Rivera K, et al. Red tape, slow emergency, and chronic disease management in post-María Puerto Rico. Crit Public Health. 2021 Nov 10; 32(4):1–14. 10.1080/09581596.2021.1998376. PMC9481060 36118129

[31] Serrano-García I. Resilience, Coloniality, and Sovereign Acts: The Role of Community Activism. Am J Community Psychol. 2020 Jan 16; 65(1–2):3–12. doi: 10.1002/ajcp.12415 31944329

[32] Pagán-Santana M, Amaya C, Rivera-Gutiérrez R, Caporali S. Chronic Diseases among Agricultural Workers in a Rural Area of Puerto Rico. J AgroMed. 2021 Apr 26; 26(2):211–219. 10.1080/1059924X.2020.1824829. 33143555

[33] censusreporter.org. Census profile: Adjuntas Municipio, PR [Internet] U.S. Census Bureau; 2021 [cited 2023 Sep 18]. Available from: http://censusreporter.org/profiles/05000US72001-adjuntas-municipio-pr/.

[34] Mariño YA, Pérez ME, Gallardo F, Trifilio M, Cruz M, Bayman P. Sun vs. shade affects infestation, total population and sex ratio of the coffee berry borer (Hypothenemus hampei) in Puerto Rico. Agric Ecosyst Environ. 2016 Apr; 222:258–66. 10.1016/j.agee.2015.12.031.

[35] censusreporter.org. Census profile: Lares Municipio, PR [Internet] U.S. Census Bureau; 2021 [cited 2023 Sep 18]. Available from: http://censusreporter.org/profiles/16000US7242498-lares-pr/.

[36] censusreporter.org. Census profile: Maricao Municipio, PR [Internet] U.S. Census Bureau; 2021 [cited 2023 Sep 18]. Available from: http://censusreporter.org/profiles/16000US7251270-maricao-pr/.

[37] censusreporter.org. Census profile: Yauco Municipio, PR [Internet] U.S. Census Bureau; 2021 [cited 2023 Sep 18]. Available from: http://censusreporter.org/profiles/16000US7288035-yauco-pr/.

[38] Andrade EL, Jula M, Rodriguez-Diaz CE, Lapointe L, Edberg MC, Rivera MI, Santos-Burgoa C. The Impact of Natural Hazards on Older Adult Health: Lessons Learned From Hurricane Maria in Puerto Rico. Disaster Med Public Health Prep. 2023 Nov 2; 7. doi: 10.1017/dmp.2021.305 34725020

[39] Buckley TD, Burnette D. Psychological sense of community, self-rated health and quality of life among older adults in Puerto Rico two years after Hurricane María. J Gerontol Soc Work. 2023 May-Jun; 66(4):512–529. 10.1080/01634372.2022.2133200. 36217794

[40] DiJulio B, Muñana C, & Brodie M. Views and experiences of Puerto Ricans one year after Hurricane Maria. [Internet]. Kaiser Family Foundation. [Cited 2024 Jun 6]. Available from: https://www.kff.org/mental-health/report/views-and-experiences-of-puerto-ricans-one-year-after-hurricane-maria/.

[41] Creswell JW, Creswell JD. Qualitative inquiry & research design: choosing among five approaches. 5th ed California: Sage Publications, Inc; 2018.

[42] Tong A, Sainsbury P, Craig J, Consolidated criteria for reporting qualitative research (COREQ): a 32-item checklist for interviews and focus groups. Int J Qual Health Care. 2007 Sep 14;19(6): 349–357. doi: 10.1093/intqhc/mzm042 17872937

[43] Nakhid C. Liming as Research Methodology, Ole Talk as Research Method—A Caribbean Methodology. Int J Educ Dev. 2019 Dec; 18(2):1–18. 10.46425/j118024995.

[44] Nakhid C, Nakhid-Chatoor M., Fernández Santana A, Wilson- Scott S. Chapter 1, Researcher Positioning in the Caribbean Research Space. In Nakhid-Chatoor M, Butcher-Lashey J, editors. Affirming Methodologies. London and New York: Routledge; 2023. p. 17–32.

[45] Saunders B, Sim J, Kingstone T, Baker S, Waterfield J, Bartlam B, et al. Saturation in qualitative research: exploring its conceptualization and operationalization. Qual Quant. 2017 Sep 14; 52(4):1893–907. doi: 10.1007/s11135-017-0574-8 29937585 PMC5993836

[46] Braun V, Clarke V. Thematic Analysis: A Practical Guide. London: Sage Publications; 2022.

[47] Azungah T. Qualitative research: deductive and inductive approaches to data analysis. Qual Res J. 2018 Nov 15; 18(4), 383–400. 10.1108/QRJ-D-18-00035.

[48] Páez Moreno R. Solidaridad relacional: una manera de abordar el derecho a la asistencia sanitaria. RevRedbioética/UNESCO. 2015 Dec; 2(12):115–25. ISSN 2077-9445.

[49] Gelpí JG. Arcadio Díaz Quiñones, El arte de bregar. [Internet]. San Juan: Ediciones Callejón, 2005. Rev. Estud. Hisp./Río Piedras. P. 437–40. Available from: https://revistas.upr.edu/index.php/reh/article/view/17749.

[50] Sasaki Y, Tsuji T, Koyama S, Tani Y, Saito T, kondo K, et al. Neighborhood Ties Reduced Depressive Symptoms in Older Disaster Survivors: Iwanuma Study, a Natural Experiment. Int J Environ Res Public Health. 2020 Jan 3; 17(1), 337. doi: 10.3390/ijerph17010337 31947798 PMC6981381

[51] Tsubouchi Y, Yorozuya K, Tainosyo A, Naito Y. A conceptual analysis of older adults’ empowerment in contemporary japanese culture. BMC Geriatr. 2021 Dec; 21(1). doi: 10.1186/s12877-021-02631-x 34852766 PMC8638333

[52] Chen J, Zhou X. Within-family patterns of intergenerational emotional closeness and psychological well-being of older parents in China. Aging Ment Health. 2020 Jan 12. doi: 10.1080/13607863.2020.1711867 31928065

[53] Schrauf R, López de Victoria Rodríguez PC. Disaster solidarity and survivor ethics: a case study of Hurricane María in Puerto Rico. Disasters, 2024 Jan; 48(1): 10.1111/disa.12593. 37227427

[54] Luo MS, Chui EW, Li LW. The Longitudinal Associations between Physical Health and Mental Health among Older Adults. Aging Ment Health. 2019 Aug 20; 24(12):1–9. doi: 10.1080/13607863.2019.1655706 31429303

[55] Fletcher PA, Worthen DL, Mcsweeney-Feld MH, Gibson A, Seblova D, Pagán L. et al. Rrual Older Adults in Disasters. Rural Older Adults in Disasters: A Study of Recovery from Hurricane Michael. Disaster Med Public Health Prep. 2022;16(6):2602–2606. 10.1017/dmp.2021.276. 34672250

[56] Kim H, Zakour M. Disaster Preparedness among Older Adults: Social Support, Community Participation, and Demographic Characteristics. J. Soc. Serv. Res. 2017 Jun 02. 43:4: 498–509. 10.1080/01488376.2017.1321081.

[57] Hamama-Raz Y, Palgi Y, Leshem E, Ben-Ezra M, Lavenda O. Typhoon survivors’ subjective wellbeing—A different view of responses to natural disaster. PLoS One. 2017 Sep 6; 12(9): e0184327. doi: 10.1371/journal.pone.0184327 28877264 PMC5587279

[58] Acosta Gelpí. El Arte de Bregar (The Art of Dealing): An Existentialism. Philosophy Study. 2023 Jul; 13(7): 303–10. doi: 10.17265/2159-5313/2023.07.005

[59] Santos-Burgoa C, Sandberg J, Suárez E, Goldman-Hawes A, Zeger S, Garcia-Meza A, et al. Differential and persistent risk of excess mortality from Hurricane Maria in Puerto Rico: a time-series analysis. Lancet Planet Health. 2018 Nov; 2(11): 478–88. doi: 10.1016/S2542-5196(18)30209-2 30318387

[60] Lebrusán I, Gómez MV. The Importance of Place Attachment in the Understanding of Ageing in Place: “The Stones Know Me”. Int J Environ Res Public Health. 2022 Dec 19; 19, 19(24): 17052. https://doi.org/10.3390/ ijerph192417052. 36554931 10.3390/ijerph192417052 PMC9779384

[61] Madera SR, Padilla M, Varas-Díaz N, Ramos-Pibernus A, González-Font Y, Santiago-Santiago A, et al. On staying: Non-migration among Puerto Rican physicians. CJMC. 2024 Jan 23; 14(2): 213–234. 10.1386/cjmc_00084_1.

[62] López-Cepero A O’Neill HJ, Marrero A, Falcon LM, Tamez M, Rodríguez-Orengo JF, et al. Association between adverse experiences during Hurricane María and mental and emotional distress among adults in Puerto Rico. Soc Psychiatry Psychiatr Epidemiol. 2022 Sep 1; 57(12):2423–32. 10.1007/s00127-022-02355-2. 36048184 PMC9434507

[63] Rodríguez-Madera SL, Varas-Díaz N, Padilla M, Grove K, Rivera-Bustelo K, Ramos J, et al. The impact of Hurricane Maria on Puerto Rico’s health system: post-disaster perceptions and experiences of health care providers and administrators. Glob Health Res Policy. 2021 Nov 10; 6:44. doi: 10.1186/s41256-021-00228-w 34753513 PMC8577961

[64] Morris ZA, Hayward RA, Otero Y. The Political Determinants of Disaster Risk: Assessing the Unfolding Aftermath of Hurricane Maria for People with Disabilities in Puerto Rico. Environ Justice. 2018 Apr; 11(2):89–94. 10.1089/env.2017.0043.

[65] Hussain B, Mirza M, Baines R, Burns L, Stevens S, Asthana S, et al. Loneliness and social networks of older adults in rural communities: a narrative synthesis systematic review. Front Public Health. 2023 May 15;11. doi: 10.3389/fpubh.2023.1113864 37255758 PMC10225733

[66] Ley Núm. 14 de 15 de marzo de 1996, según enmendada, “Ley Especial para el Desarrollo de Castañer 1996 (Cwlth) [Internet]. Oficina de Gerencia y Presupuesto. [cited 2024 Jul 6]. Available from: https://bvirtualogp.pr.gov/ogp/Bvirtual/leyesreferencia/PDF/Incentivos/14-1996/14-1996.pdf.

[67] Adelman RD, Tmanova LL, Delgado D, Dion S, Lachs M. Caregiver Burden A Clinical Review. JAMA. 2014 Mar 12; 311(10): 1052–60. doi: 10.1001/jama.2014.304 24618967

[68] Mackey Ian. PAT [Internet]. portable atlas version 1.1; 2013 [cited 2024 Jul 10]. Available from: https://ian.macky.net/pat/index.html.

