# Supplementary material for: “Many old people taking care of old people”: Experiences of older adults after Hurricane María in Puerto Rico
Source: PLoS One. 2025 Jan 16;20(1):e0316156. doi: 10.1371/journal.pone.0316156 (PMC11737680; doi:10.1371/journal.pone.0316156)
Supplement: S1 Text — (DOCX) [file pone.0316156.s001.docx]

**S1 Table. Consolidated criteria for reporting qualitative studies (COREQ): a 32-item checklist.**

| **No. Item** | **Guide questions/description** | **Reported on Page #** |
| --- | --- | --- |
| **Domain 1: Research team and reflexivity** | | |
| *Personal Characteristics* | | |
| 1. Interviewer/facilitator | Which author/s conducted the interview or focus group? | Page 11 |
| 2. Credentials | What were the researcher’s credentials? *E.g. PhD, MD* | Page 8 |
| 3. Occupation | What was their occupation at the time of the study? | Page 8 |
| 4. Gender | Was the researcher male or female? | Page 8 |
| 5. Experience and training | What experience or training did the researcher have? | Page 8 |
| *Relationship with participants* | | |
| 6. Relationship established | Was a relationship established prior to study commencement? | Page 8 |
| 7. Participant knowledge of the interviewer | What did the participants know about the researcher? *e.g. personal goals, reasons for doing the research* | Page 8 |
| 8. Interviewer characteristics | What characteristics were reported about the interviewer/facilitator? *e.g. Bias, assumptions, reasons and interests in the research topic* | Page 8 |
| **Domain 2: study design** | | |
| *Theoretical framework* | | |
| 9. Methodological orientation and Theory | What methodological orientation was stated to underpin the study? *e.g. grounded theory, discourse analysis, ethnography, phenomenology, content analysis* | Page 12 |
| *Participant selection* | | |
| 10. Sampling | How were participants selected? *e.g. purposive, convenience, consecutive, snowball* | Page 10 |
| 11. Method of approach | How were participants approached? *e.g. face-to-face, telephone, mail, email* | Pages 10 & 11 |
| 12. Sample size | How many participants were in the study? | Page 9 |
| 13. Non-participation | How many people refused to participate or dropped out? Reasons? | Page 11 |
| *Setting* | | |
| 14. Setting of data collection | Where was the data collected? *e.g. home, clinic, workplace* | Page 11 |
| 15. Presence of non-participants | Was anyone else present besides the participants and researchers? | Page 11 |
| 16. Description of sample | What are the important characteristics of the sample? *e.g. demographic data, date* | Pages 9 |
| *Data collection* | | |
| 17. Interview guide | Were questions, prompts, guides provided by the authors? Was it pilot tested? | Page 11 |
| 18. Repeat interviews | Were repeat interviews carried out? If yes, how many? | N/A |
| 19. Audio/visual recording | Did the research use audio or visual recording to collect the data? | Page 11 |
| 20. Field notes | Were field notes made during and/or after the interview or focus group? | N/A |
| 21. Duration | What was the duration of the interviews or focus group? | Page 7 |
| 22. Data saturation | Was data saturation discussed? | Pages 12 |
| 23. Transcripts returned | Were transcripts returned to participants for comment and/or correction? | N/A |
| **Domain 3: analysis and findings** | | |
| *Data analysis* | | |
| 24. Number of data coders | How many data coders coded the data? | Page 12 |
| 25. Description of the coding tree | Did authors provide a description of the coding tree? | N/A |
| 26. Derivation of themes | Were themes identified in advance or derived from the data? | Page 12 |
| 27. Software | What software, if applicable, was used to manage the data? | N/A |
| 28. Participant checking | Did participants provide feedback on the findings? | N/A |
| *Reporting* | | |
| 29. Quotations presented | Were participant quotations presented to illustrate the themes / findings? Was each quotation identified? *e.g. participant number* | Pages 11-22 |
| 30. Data and findings consistent | Was there consistency between the data presented and the findings? | Pages 13-22 |
| 31. Clarity of major themes | Were major themes clearly presented in the findings? | Pages 13-14 |
| 32. Clarity of minor themes | Is there a description of diverse cases or discussion of minor themes? | N/A |

Developed from:

Tong A, Sainsbury P, Craig J. Consolidated criteria for reporting qualitative research (COREQ): a 32-item checklist for interviews and focus groups. International Journal for Quality in Health Care. 2007. Volume 19, Number 6: pp. 349–357.
