# Supplementary material for: “Many old people taking care of old people”: Experiences of older adults after Hurricane María in Puerto Rico
Source: PLoS One. 2025 Jan 16;20(1):e0316156. doi: 10.1371/journal.pone.0316156 (PMC11737680; doi:10.1371/journal.pone.0316156)
Supplement: S2 Text — (DOCX) [file pone.0316156.s002.docx]

Participant code: __________

Semi-structured interview guide

Date: ______________________

Place of interview: ______________________

Sector/Neighborhood: ______________________

Interviewer: ______________________

Instructions:

Section 1 – Demographic questions

1. What year were you born in?
2. Where were you born?
3. What is your gender?
4. Where do you currently live?
5. With whom do you currently live?
6. Are you the proprietor of your current home?
7. What is your marital state?
8. Do you have children? If so, how many?
9. What is the highest level of education you have achieved?
10. What do you dedicate your time to (i.e. caregiving, working, etc.)?
11. Do you currently have a job?
12. What type of job do you conduct?
13. In what area of Puerto Rico do you do your job?
14. Approximately, how much do you earn monthly?
15. Do you have medical insurance? Private or public?
16. Were you diagnosed with any physical health condition before hurricane Maria? If so, which one? – What about after the hurricane?
17. Do you currently use any medication for your physical health condition?
18. Were you diagnosed with any mental health condition before hurricane Maria? If so, which one? – What about after the hurricane?
19. Do you currently use any medication for your mental health condition? Section 2 – General questions about the hurricanes
20. What did the hurricanes Irma and Maria meant for you?
21. What challenges did you personally experience during and after both hurricanes?
22. How do you believe the hurricane impacted you? How about your family? How about your community? How about the town?
23. What do you think have been the challenges people in Adjuntas/Castañer has faced to recover from the hurricanes?
24. How would you consider these challenges have been different to those faced by other municipalities in urban areas?

Section 3 – Questions about emotional well-being (mental health) and its management

1. What do you think were the main challenges to the emotional wellbeing (mental health) of people 60 years or older during and after the hurricane?
2. How did you experience these challenges?
3. How much did these challenges last? How did this make you feel?
4. What did you do to successfully recover or adapt to these challenges?
5. What role did: (Ask about: the government, hospitals, pharmacies, the community, community organizations, insurance companies, churches) play in attending these challenges for people 60 years or older? Provide specific examples.
6. How do you think your emotional wellbeing (mental health) changed after the hurricane?
7. What changes have you implemented to manage these challenges in the future?
8. What changes do you believe the government/previously mentioned organizations have to implement to attend the emotional and mental health challenges people 60 years or older face during disaster situations?

Section 4 – Questions about the physical and social environment

1. How did the environment in the area where you live changed after the hurricane?
2. How did you react to the damage the hurricane caused to the environment and other structures? How did it make you feel? How do you believe it had an impact on your wellbeing?
3. How have these changes impacted the way in which you relate to people in your community, caregivers and health professionals?
4. How has the recovery process been (e.g. road and power repairs)? Who has been mainly involved in the repair process (e.g. government, community organizations)? Were you involved in any way? How did that make you feel?
5. Talk to me a little about how your personal activities were affected after the hurricane (e.g. job, health services, transportation, food, exercise).
6. From your point of view, how was the community response after the event?
7. Talk to me a little about how your family structure and your community changed after the event. Did any family members or neighbors move outside of Puerto Rico? Did any family members who were living outside the island return? Did any other family members come to live at your home? How did these changes make you feel?

Are there any other aspects that you believe we should know about concerning the mental health of people 60 years or older after a natural disaster?

Thank you for your time.
