# Supplementary material for: “Many old people taking care of old people”: Experiences of older adults after Hurricane María in Puerto Rico
Source: PLoS One. 2025 Jan 16;20(1):e0316156. doi: 10.1371/journal.pone.0316156 (PMC11737680; doi:10.1371/journal.pone.0316156)
Supplement: S3 Text — (DOCX) [file pone.0316156.s003.docx]

**Translated quotes**

**Theme one: “Many old people taking care of old people” - Challenges to wellbeing after a natural hazard among older adults in Adjuntas and Castañer, PR**

- “There are many old people taking care of old people, that is, and… there are many, when they do not have adequate resources, an old person taking care of another old person is not the best thing, right.”
- “Aquí hay muchos viejos cuidando de viejos, o sea y… hay muchos, cuando no tienen unos recursos adecuados, un viejo cuidando de otro viejo no es lo mejor, verdad.”; (Professional, Administrator of local hospice program)
- “This is very difficult, well… and then just being alone here, well, with... Not entirely alone because my brother is here, but he tends to go out and so on. But this, to live such a situation alone. Washing clothes by hand, with a bedridden person, you know, that’s been very difficult.”
- “No... bien difícil este, bien... y después uno solo aquí pues con el… Solo no porque esta mi hermano, pero como ellos cogen calle y demás pues. Pero este, uno pasar solo este una situación así. Lavando ropa a mano, con una persona encamada, sabes eso era bien difícil.” (Female denizen of 71 years old; wife and caretaker)
- “... they live alone, you know, the children, the fam[ily], they forgot, they went, and these People live alone.”
- “… viven solos, sabes, los hijos, los fam[miliares], se olvidaron, fueron y estas personas viven solitas.” (Female denizen of 60 years, Alzheimer’s patient caretaker)
- “[B]ecause we lost practically everything we had. The roof and everything flew out, everything went flying and that's traumatizing. So since you are not wealthy, you do not have money, you have to wait for donations…”
- “[P]orque perdimos todo lo que teníamos prácticamente. El techo y todo se fue, todo salió volando y eso es traumatizante. Entonces como uno no es pudiente, no tiene dinero, tiene que esperar que le donen…” (Male denizen of 68 years)
- “They are alone, and their children want to send for them, but they don’t want to leave the island. It’s the same as with the hurricane, they didn’t want to leave the house.”
- “Son viejos solos, y los hijos los quieren mandar a buscar, pero ellos no quieren dejar la Isla. Es lo mismo que con el huracán, que no querían dejar la casa.” (Female denizen of 81 years)
- “I can tell you that most of who lived through that experience are no longer here. They already died in the program. And many of those were because of sadness, they were very sad.”
- “Yo te puedo decir que la mayor parte de lo que vivieron esa experiencia ya no están, ya murieron en el programa. Y muchos de esos fue por tristeza, les dio mucha tristeza.” (Social Worker for Hospice)

**Theme 2: “In this community each one was working together”—Relational Solidarity among older adults in Adjuntas and Castañer**

- “… the commitment that we had as citizens of here of Castañer. The union, the commitment…the familiarity because everyone here practically, although we were not family, since we were all always familiar with what do you need, if I go someplace to buy something, “what do I bring to you”, “what do I give to you”. That. Mainly among everything was the union we had, but the commitment. To be aware of everyone...
- “…el compromiso que nosotros teníamos como ciudadanos de aquí de Castañer. La unión, el compromiso, este… la familiaridad porque aquí todos prácticamente, aunque no éramos familia, pues siempre estábamos todos familiarizados con qué tú necesitas, si yo voy para algún sitio a comprar algo, qué te traigo, qué te llevo. Eso. Principalmente entre todo fue la unión que tuvimos, pero el compromiso. Estar pendiente de todas las personas... (Female Caregiver feminine of 61 years)
- ““…I’m proud to belong to Portillo, right, to Tanamá…”
- “orgullosa de pertenecer a Portillo, verdad,” (female denizen of 60 years old, caregiver of Alzheimer patients).
- “…[m]ovement from the government was very slow and that forced people to come together to work, everyone in their respective area.”
- “…[m]overse del gobierno fue muy lento y eso obligó a las personas a unirse para bregar cada cual en su área.” (Male denizen of 68 years).
- “It took about four months after Hurricane María, if not more, for a mayor to come here. No one from the government showed up here, nothing.”
- “Pasaron como cuatro meses después del huracán María, si no más, para que viniera un alcalde aquí. Aquí no se presentó nadie de gobierno, nada.” (Female caregiver of 61 years)
- “We formed a team of doctors, mental health, and nursing staff, and we began to visit shelters. It could be said that we had the tools to know what was going to be done there, because there was no plan” (Castañer male clinical psychologist)
- “Se hizo un equipo de médicos, salud mental y de enfermería, y entonces se comenzaron a visitar los refugios. Se podría decir que teníamos las herramientas de saber cómo era lo que se iba hacer allí porque no había un plan.” (Castañer male clinical psychologist)
- “The Red Cross, the National Guard did come a lot, many people came here personally and one of these came from institutions, they also came. And many people here still bring things to this date.”
- “La cruz roja, la guardia nacional si vinieron mucho, aquí personalmente vinieron estas muchas personas y de instituciones está este de estas también vinieron. Y muchas personas, aquí todavía traen cosas a esta fecha.” (Female denizen of 71 years).
- “It was difficult… But the indifference here in Adjuntas is also difficult… Nobody, nobody here. My neighbors, I've been here for about 39 years more or less and my neighbors, some came later, but there were some who had been here for a long time... [and were] quite indifferent."
- “Era difícil… Pero también es difícil la indiferencia que hay aquí en Adjuntas…Nadie, nadie aquí. Mis vecinos, yo llevo aquí como 39 años más o menos, y los vecinos míos llegaron unos después, pero habían unos que estaban hace tiempo… bien indiferentes.” Female denizen of 80 years).
- “…in this community they were well united. In this community each one was working together.”
- “…pero en esta comunidad sí estuvieron bien unidos. En esta comunidad era mano a mano cada uno” (Female denizen of 78 years).

**Theme 3: “If I decay, I am not going to solve anything…” - *La brega*** **among older adults in Adjuntas and Castañer**

- “The whole house destroyed, and… he told me; ‘We are alive, we have to push forward’. And my wife who is strong, gave me her support, she would always tell me; ‘we are going to *bregar*, forget about it, little by little’. And my roof had plastic coverings for more than a year...”
- “Toda la casa destruida y yo dije, me dijo; ‘Estamos vivos, hay que echar pa’ lante’. Y mi esposa que es fuerte me, me dio su apoyo, siempre me decía; ‘vamos a bregar, olvídate, poco a poco’. Y yo estuve con toldos más de un año, más de un año estuve con toldos.” (Male denizen of 68 years)
- “…I would look for a candle, lit it and with that we would light up around. And to cook, that we couldn’t, well we would go downstairs too. Should we run out of gas, we would go down there to the kitchen. Turn on a little fire over there and yes, [I] *bregaba* downstairs, cooking and everything.”
- “Buscaba una vela, la prendía y con eso pues nos alumbrábamos y para cocinar que no podíamos y eso pues nos íbamos a cocinar abajo también. Que se nos acabara el gas íbamos allá abajo a la cocina. Préndete un fogoncito por allí y si, bregaba abajo, cocinaba y todo.” (Female denizen of 80 years)
- “So, well, how do I put it, I am human, like, I do not give up, I am not insensitive, but I remain firm, but always seeing and visualizing that if I decline, I’m not going to solve anything, that I need to draw strength from myself, strength from wherever I have to look for it, to face whatever comes and contribute what I can.”
- “Así pues, como te digo, yo soy humana, como que, yo no decaigo, no soy insensible, pero me mantengo firme pero siempre viendo y visualizando que con yo decaer no voy a resolver, que yo necesito sacar fuerzas de mí, fuerzas de donde haya que buscarla para enfrentar lo que sea y aportar lo que pueda.” (Female denizen of 78 years)
- “…Well, because you have the people, because you have the infrastructure, because you have the relationships, because you have, in the case of Casa Pueblo it has credibility and a history. When we face problems, we face them [with our] heads up high and we solve them with what’s within our reach, with the limitations that one might have."
- “Bueno, porque tienes la gente, porque tienes la infraestructura, porque tienes las relaciones, porque tienes en el caso de Casa Pueblo tiene una credibilidad y una historia cuando enfrentamos problemas los atendemos de frente y los pues lo resolvemos con lo que está al alcance con las limitaciones que uno pueda tener. (Casa Pueblo-Community-based organization from Adjuntas)
- “…since I quickly got involved in working, maybe I didn't have a lot of time to regret and not because I wasn't emotionally upset, it was just time to get involved, to do what I had to do.”
- “…como rápido me involucré en trabajar quizás no tuve mucho tiempo para lamentaciones y no porque no emocionalmente no estaba afligida, es que era tiempo de meter mano, de hacer lo que tenía que hacer.” (Female psychologist)
